# SP3-induced Timeless transcription contributes to cell growth of lung adenocarcinoma cells

**DOI:** 10.1371/journal.pone.0298295

**Published:** 2024-02-14

**Authors:** Ping Tian, Dajun Du, Li Yang, Nan Zhou, Ling Tao

**Affiliations:** 1 Medical School, Xinyang Vocational and Technical College, Xinyang, Henan, China; 2 Department of Surgical Oncology, Xinyang Central Hospital, Xinyang, Henan, China; 3 Inspection School, Xinyang Vocational and Technical College, Xinyang, Henan, China; 4 Department of Medical Oncology, Xinyang Central Hospital, Xinyang, Henan, China; BMSCE: BMS College of Engineering, INDIA

## Abstract

**Background:**

Timeless is well-known for its key role in replication checkpoints. Recent studies reveal the involvement of Timeless and specificity protein (SP) 1 in human malignancies. However, no evidence proved the interaction between SP3 and Timeless in lung adenocarcinoma (LUAD).

**Methods:**

The expression and clinical significance of Timeless were analyzed using the LUAD dataset downloaded from the Cancer Genome Atlas (TCGA). Lentivirus-mediated Timeless knockdown in A549 cells was used to examine the role of Timeless in cell proliferation and pemetrexed (PEM) resistance. Transcription factors (TFs) bound to the Timeless promoter were identified by DNA pull-down technology with HPLC-MS/MS analysis and analyzed by the Kyoto Encyclopedia of Genes and Genomes (KEGG) pathway. Dual-luciferase reporter assay was used to determine the activity of SP3 in Timeless transcription.

**Results:**

Timeless was overexpressed in LUAD samples, and it could serve as a potential diagnostic or prognostic biomarker for LUAD patients. shTimeless-mediated knockdown of Timeless reduced cell viability and proliferation and sensitized PEM-resistant A549 cells to PEM. Four fragments (F1: 1–373 bp), (F2: 374–962 bp), (F4: 1274–1645 bp), and (F5: 1646-2000bp) were confirmed as the TF binding profiles of the Timeless promoter. KEGG analysis showed that the TFs bound to the Timeless promoter had relevance to spliceosome, RNA transport, and mRNA surveillance pathways. SP3 promoted the transcription of Timeless via the F2 fragment (374–962 bp) binding motif.

**Conclusion:**

Upregulation of Timeless mediated by SP3 promotes LUAD cell proliferation, providing evidence to support that targeting the SP3/Timeless axis may be a potential therapeutic strategy against LUAD.

## Introduction

As a common pathological type of non-small cell lung cancer (NSCLC), lung adenocarcinoma (LUAD) is mostly located in the bronchial mucosal epithelium [1]. Typical symptoms of the disease are hemoptysis, cough, and shortness of breath. LUAD is mainly caused by various factors, such as long-term smoking, environmental pollution, and occupational exposure [2]. Because of nonspecific clinical symptoms, most patients with LUAD have progressed to an intermediate or advanced stage at the time of initial diagnosis [3]. Despite improvements in surgery, radiotherapy, chemotherapy, and targeted therapy, the prognosis for lung cancer patients remains suboptimal, with a five-year survival rate of about 10%-20% [4]. Pemetrexed (PEM) is an anti-tumor drug that blocks the release process of enzymes required for purine and pyrimidine synthesis, thereby sustaining cell division activities in the S phase and significantly inhibiting the growth of cancer cells [5]. PEM has been approved as a first-line chemotherapy agent for non-squamous NSCLC [6]. PEM is used in patients with LUAD who cannot receive targeted therapy due to its low toxicity and low adverse effects. Moreover, it is more suitable for older patients with poor general conditions and has shown advantages in second-line therapy after failure of EGFR-tyrosine kinase inhibitor treatment [7, 8]. However, acquired resistance to PEM frequently occurs after use for approximately 2–5 months. Therefore, it is particularly important to unveil the mechanism of PEM resistance development in LUAD.

The circadian clock is a stable intrinsic biorhythm formed by organisms over a long period of evolution, which is regulated at the molecular level by many circadian clock genes [9]. The coordinated expression of circadian clock genes regulates the growth and aging of cells and organs through metabolism [10]. The occurrence of human tumor is due to the loss of normal regulation of cell growth [11]. Increasing evidence confirms that circadian clock genes play a direct or indirect role in tumor initiation and progression by regulating cell biological processes and behaviors [12]. The Timeless gene, one of the circadian clock genes, is located on chromosome 12 of humans, with a total length of about 43 kb [13]. The Timeless protein encoded by the Timeless gene consists of 1208 amino acid residues, and it actively participates in the regulation of circadian rhythms by forming dimers with CRYPTOCHROME and PERIOD proteins. It has been confirmed that the Timeless protein is involved in maintaining cell cycle, increasing DNA polymerase activity, and maintaining telomere length during DNA damage [14, 15]. Alteration in Timeless expression can play an important role in tumorigenesis and cancer development. Upregulation of Timeless has been detected in various cancers, including lung cancer [16], breast cancer [17], and colorectal cancer [18], and its high expression predicts poor survival outcomes. Timeless is capable of conferring cisplatin resistance in nasopharyngeal and cervical cancers [19, 20]. Furthermore, Timeless has been identified as a key player for NSCLC development [21], and its expression is associated with progression-free survival in lung cancer patients [22]. However, the exact role and mechanism of Timeless in LUAD are unclear.

Specificity protein (SP) family of transcription factors (TFs) only binds to cis-regulatory elements in their target promoters, thus enhancing or inhibiting gene expression [23]. SP family, as a type of special TFs, contains highly conserved DNA-binding regions and three tandem Cys2His2 zinc finger domains at the carboxyl end, which can bind to GC (GGGGCGGGG)/GT (GGTGTGG) boxes and basic transcription elements [24]. Among them, the DNA binding region sequences of SP1 and SP3 proteins have more than 90% homology, and the binding sequence GC/GT boxes of SP1 and SP3 are often distributed in the promoter or enhancer of genes in the form of multiple copies. SP3 has been unveiled to exert pro-oncogenic functions in human carcinogenesis [25]. Studies have revealed that SP1 is involved in the regulation of Timeless expression in breast cancer [26, 27]. However, it is unclear whether SP3 interacts with Timeless to affect the progression of LUAD.

Therefore, we aimed to investigate whether SP3 participates in PEM resistance in A549 cells by regulating the expression of Timeless. This study preliminarily investigates the effect of TF SP3 on the transcriptional activity of Timeless, providing a rationale for the regulatory network and mechanism of SP3 in LUAD.

## Materials and methods

### Data collection and processing

The RNA-seq transcriptome data and clinical data were downloaded from the Cancer Genome Atlas (TCGA) database (http://www.cancergenome.nih.gov/). The differences in Timeless transcripts between LUAD samples and paired non-tumor samples (n = 58) were analyzed using stats [4.2.1] and car packages, and the data were visualized using the ggplot2 package [3.3.6]. For the receiver operating characteristic (ROC) analysis, the clinical data of LUAD samples (n = 539) and normal samples (n = 59) were downloaded. The pROC package [1.18.0] was used for data analysis, and the results were visualized using ggplot2 [3.3.6]. For OS analysis, the clinical data of LUAD patients (n = 500) were downloaded. The survival package [3.3.1] was used for proportional risk hypothesis testing and fitted survival regression, and the results were visualized using the survivor package and ggplot2 package [3.3.6]. A search for sequence (~ 2000 bp) of human Timeless promoter (NM_003920.5) was performed using the Ensemble database (http://uswest.ensembl.org/index.html). Prediction of TF binding profiles of the Timeless promoter was performed through the JASPAR database (https://jaspar.genereg.net/).

### Cell line and culture conditions

Human A549 cells (Cat#CCL-185™, ATCC, Manassas, VA, USA) and H1299 cells (Cat#CL-0165, Procell, Wuhan, China) were maintained at 37°C in the humidified incubator with 95% relative humidity and 5% carbon dioxide. The medium for cell culture was Kaighn’s Modification of Ham’s F-12 Medium (F-12K medium, for A549) (Cat#30–2004, ATCC) or RPMI-1640 (Cat#G4538, Servicebio, Wuhan, China), supplemented with 10% fetal bovine serum (FBS) (Cat#G8001, Servicebio) and 1% antibiotics (Cat# PB180120, penicillin/streptomycin, Procell). HEK293T cells (Cat#CL-0005, Procell) were maintained using standard protocols (DMEM+10% FBS+1% penicillin/streptomycin) provided by Procell.

Pemetrexed (PEM, Cat#SML1490) was obtained from Sigma (St. Louis, MO, USA). PEM-resistant A549 (A549/PEM) cells were established by exposing the parental cell line to a single high concentration of PEM (the 50% inhibitory concentration (IC50) of PEM for A549 cells) over 6 months [28]. The A549/PEM cells were confirmed to get stable resistance by calculating the PEM resistant index (RI) using the method: RI = the IC50 value of A549/PEM cells/the IC50 value of A549 cells. When the RI > 5, the cells were identified to have the phenotype of PEM resistance. After cultivation in a low concentration (0.5 μM) PEM medium, A549/PEM cells were used for subsequent experiments.

### Lentiviral transduction

The short hairpin RNA (shRNA) targeting Timeless was synthesized by Ribobio (Guangzhou, China). To knock down Timeless in A549 and H1299 cells, the lentiviral vectors against Timeless (shTimeless, 5’-CCGGGCCCACACTAACCATTGCATTCTCGA-3’) or pLKO.1-puro shRNA (shNC) (Cat#30323, Addgene, Cambridge, MA, USA), as well as packaging vectors pMD2G (Cat#12259, Addgene) and psPAX2 (Cat#12260, Addgene), were transfected into 293T cells (Cat#CRL-3216, ATCC) by using the iMFectin PolyDNA Transfection Reagent (Cat#I7100-100, GenDepot, Barker, TX, USA) following the manufacturer’s suggested protocols. Forty-eight hours, viral supernatant fractions were collected and filtered through a 0.45 μm syringe filter (the transducing unit of the generated virus was 2.5 × 10^7^ TU/mL). A549 and H1299 cells were infected with lentiviruses with 10 multiplicity of infection (MOI) in media containing 10 μg/mL polybrene (Cat#TR-1003, Sigma). The medium was replaced with a fresh complete growth medium containing 2 μg/mL puromycin (Cat#540222, Sigma) at 16 h after infection, and the virus-positive cells were selected by 2 μg/mL puromycin for 21 days.

### Overexpression of SP3

Human SP3 expression plasmid pLV3-CMV-SP3 (Cat#540222, VT033149) was procured from Solarbio (Beijing, China), and pLV3-NC (vec) was used as a control. For transfection, when cell fusion reached 80%-90%, a mixture of 3 μg plasmids and 5 μL RFect plasmid DNA transfection reagent (Cat#21015, Baidai, Changzhou, China) was added to each well.

### Real time-quantitative PCR (RT-qPCR)

Lentivirus-infected or un-transduced LUAD cells were transfected with SP3 expression plasmid or vector control for 48 h. Extraction of total RNA from cultured cells was carried out using the RNeasy Mini kit (Cat#74106, Qiagen, GmBH, Germany). Production of complementary DNA was performed with extracted total RNA using a high-capacity complementary DNA reverse transcription kit (Cat#4368814, Thermo, Waltham, MA, USA). Quantitative PCR was performed on a real-time PCR system using TaqMan Fast Advanced Master Mix (Cat#4444963, Thermo) with gene-specific primers (S1 Table). Relative quantification was standardized to β-actin and determined using the 2^-ΔΔCt^ method [29].

### 5-Ethynyl-2’deoxyuridine (EdU) assay

The proliferative capacity of lentivirus-infected LUAD cells transfected with or without SP3 expression plasmid or vector control for 48 h was analyzed according to the manufacturer’s instructions of the Cell-lightTM EdU DNA cell proliferation kit (Cat#C10310-3, Ribobio). Briefly, LUAD cells (1×10^4^) were seeded into 96-well plates. On the second day, the cells were incubated with 10 μM EdU for 2 h. After fixation with 4% paraformaldehyde (Cat#P1110, Solarbio), the cells were stained with Apollo Dye Solution. The nuclei of the cells were stained with Hoechst 33342. Image acquisition was performed using an Olympus SZH10 Stereo microscope (Olympus, Tokyo, Japan).

### Cell counting kit-8 (CCK-8) assay

Analysis of cell viability was executed in conformity with the manufacturer’s instructions using the cell proliferation and cytotoxicity assay kit (Cat#CA1210, Ribobio). Briefly, lentivirus-infected A549, H1299 and A549/PEM cells transfected with or without SP3 expression plasmid or vector control for 48 h (1×10^4^) were plated into the 96-well plates. Forty-eight hours later, 10 μL of the CCK-8 solution was added to each well. After incubation for 2 h, the optical density (OD) values were determined at the wavelength of 450 nm under a microplate reader (BioTek, Winooski, VT, USA). For the half-maximal inhibitory concentration (IC50) of PEM, the examined cells were exposed to various concentrations of PEM (0, 0.5, 1, 2, 4, 8, 16, 32 μM) for 72 h. The IC50 values were calculated using the Graphpad prism software version 8.0.2. (Graphpad Prism, La Jolla, CA, USA).

### Immunofluorescence

Lentivirus-infected A549 and H1299 cells transfected with or without SP3 expression plasmid or vector control for 48 h were cultured on dishes for 24 h. For immunofluorescence staining, the cells were incubated with a primary antibody against PCNA (Cat#ab92552, 1:250, Abcam, Cambridge, MA, USA) at 4°C overnight after being fixed with 4% paraformaldehyde for 20 minutes and permeated with 0.5% Triton X-100 (Cat#P1080, Solarbio) for 30 minutes. Then, the cells were incubated with the Alexa Fluor^®^ 488 goat anti-rabbit antibody (Cat#ab150077, 1:1000, Abcam) at room temperature. Staining of the nuclei was performed by 4’,6-diamidino-2-phenylindole (DAPI) (Cat#D9542, Sigma) incubation. Representative images were taken using an Olympus FV10-FWS confocal microscope.

### DNA pull-down assay and proteome analysis

The synthesis of the sequence (2000 bp) for the human Timeless promoter was done by Genecreate (Wuhan, China). Biotin-labeled primers (S1 Table) were used to amplify four Timeless promoters with different lengths, including one promoter with a full-length sequence P0 (0-2000bp) and three truncated promoters P1 (1-1184bp), P2 (374-1645bp), and P3 (823-2000bp). The production of PCR amplification was validated by 1.5% agarose gel electrophoresis (Cat#G-10, Biowest, Loire Valley, France). The nucleoprotein of A549 cells was extracted with the nuclear protein extraction kit (Cat#RY3001, Henan Writegene Biotechnology CO., LTD., Zhengzhou, China). The DNA pull-down was performed with a DNA pull-down test kit (Cat#RY5003), according to the manufacturer’s protocols (Henan Writegene Biotechnology CO., LTD.). In brief, biotin-labeled DNA sequence probes P0, P1, P2, or P3 (10 μg) were dissolved in 400 μL of 2× binding&washing buffer, respectively, followed by co-incubation with BeyoMag™ streptavidin magnetic beads (35 μL) (Cat#P2151, Beyotime, Shanghai, China). After 30 min, the probe-bead complex was incubated with the nucleoprotein of A549 cells for 1 h. Bound proteins were eluted with the protein eluent, followed by boiling for 5 min in the SDS loading buffer. The harvested protein in each group was subsequently processed for qualitative proteome analysis by Qinglianbio Biotechnology Co., Ltd. (Beijing, China) with RIGOL L-3000 HPLC System (RIGOL, Beijing, China). The raw data were searched against the homo sapiens database by the Proteome Discoverer 2.4 with Sequest HT (Thermo).

### Pathway enrichment analysis

The proteins exhibiting differential binding to the biotin-labeled promoter probe and negative control were performed the Kyoto Encyclopedia of Genes and Genomes (KEGG) pathway analysis at https://www.kegg.jp/.

### Dual-luciferase reporter assay

The Timeless promoter sequence was ligated into the pGL3-basic vector (Promega, Madison, WI, USA) to create the Timeless-Luc vector after restriction digestion. For the luciferase reporter assay, A549 and H1299 cells were plated in 24-well plates at 1 × 10^5^ cells per well 24  h before transfection of luciferase reporter plasmids. A mixture of luciferase plasmids Timeless-Luc (200 ng), pRL-TK vectors (50 ng) (Promega), and SP3 overexpression plasmids or NC was transfected into A549 and H1299 cells. Forty-eight hours later, the luciferase activities of Firefly and *Renilla* were measured by Synergy Neo2 HTS Multi-Mode Microplate Reader (Biotek, Winooski, VT, USA) according to the manual of dual-luciferase reporter assay system (Cat#E1910, Promega). Firefly luciferase activity was normalized to *Renilla* activity.

### Chromatin immunoprecipitation (ChIP) assay

ChIP experiments were carried out using a ChIP^TM^ Enzymatic Assay Kit (Cat#P2083S, Beyotime). Briefly, A549 cells were fixed with 1% formaldehyde and incubated with glycine reagent. Cells were then washed with PBS plus inhibitors of phosphatase and protease, and followed by the preparation of cell nucleus. For DNA fragmentation, the MNase was subsequently used according to the Kit protocols. Following the fracture of nuclear membrane, total extractions were incubated with anti-SP3 (Cat#ab227856, 1:300, Abcam) or anti-IgG isotype (Cat#ab172730, 1:500, Abcam) antibody and Protein A/G Magnetic Beads overnight at 4°C. Using the DNA purification Kit (Cat#D0033, Beyotime) to purify the bead-bound DNA, qPCR was used to detect the enrichment level of the Timeless promoter containing the F2 fragment using specific primer sets shown in S1 Table.

### Western blotting

LUAD cells were transfected with SP3 expression plasmid or vector control for 48 h. For the preparation of whole cell lysates, transfected cells were washed with ice-cold PBS and then lysed in RIPA lysis buffer containing protease inhibitor cocktail (Cat#P8340, Sigma) and phosphatase inhibitor cocktail (Cat#P0044, Sigma). Lysates were cleared by centrifugation (13,300 rpm) at 4°C. Protein concentration was determined using the BCA assay (Cat#23225, Thermo) in flat-bottom 96-well plates according to the manufacturer’s protocols. Proteins were separated by SDS-PAGE and transferred to a nitrocellulose membrane (Cat#10600007, GE Healthcare, Logan, UT, USA). The following antibodies were used for protein detection: Timeless (Cat#ab109512, 1:50000, Abcam), β-actin (Cat#ab124964, 1:1000, Abcam), and goat anti-Rabbit IgG H&L (HRP) (Cat#ab97051, 1:10000, Abcam). Protein bands were visualized using Western Lightning Plus-ECL (Cat#NEL103001EA, PerkinElmer) according to the manufacturer’s instructions and developed using a ChemiDoc MP Imaging System (Bio-Rad). The raw uncropped images of all blots were shown in S1 Raw images.

### Statistical analysis

All experiments were performed at least three times. All statistical data were calculated using GraphPad Prism 8. Results were expressed as means ± standard deviation. Significance was analyzed by a two-tailed unpaired Student’s *t*-test, one-way ANOVA followed by Tukey’s comparisons test or two-way ANOVA followed by Holm-Sidak’s multiple comparison tests. *P* values of <0.05 were considered significant.

## Results

### Timeless has a potential diagnostic and prognostic value for LUAD

**Fig 1:**
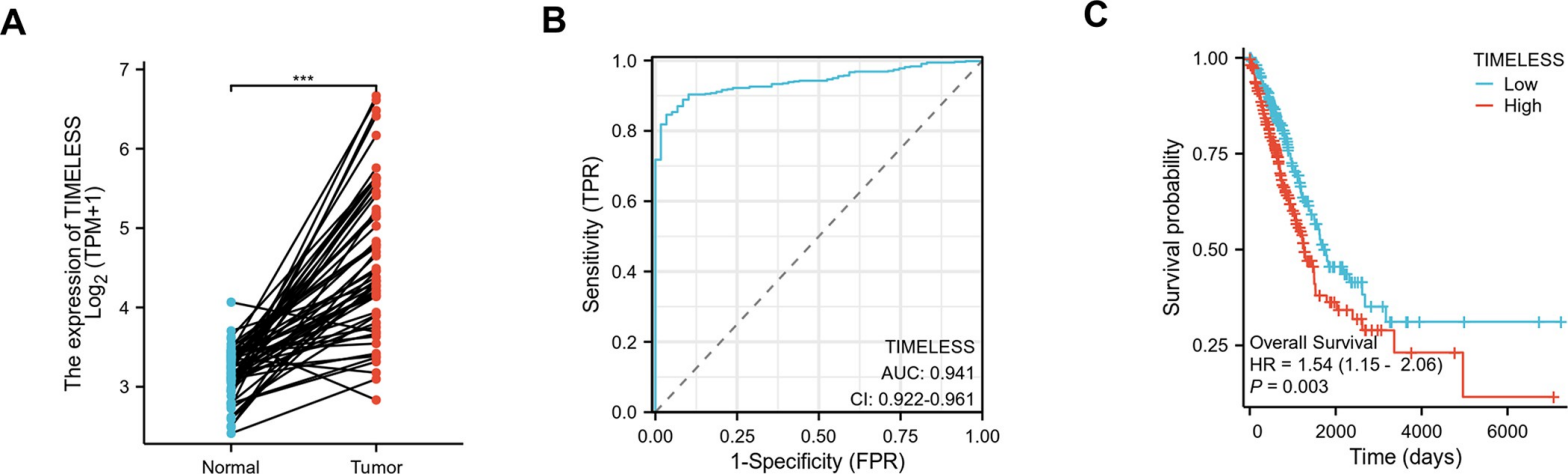
Alterations in the expression of Timeless in LUAD and its clinical significance. (A) The expression of Timeless in LUAD samples and paired normal samples in TCGA-LUAD. (B) ROC curves measuring the sensitive and specific values of Timeless in TCGA-LUAD. (C) Kaplan-Meier curves of patients with high and low Timeless in TCGA-LUAD (log-rank test). *** *p* < 0.001.

To study the alteration in the transcription level of Timeless, we downloaded the RNA-seq data related to LUAD from the TCGA database. Our analysis showed a marked elevation in Timeless expression in LUAD samples in comparison with their non-tumor counterparts (Fig 1A). The ROC curves were utilized to make an evaluation of the diagnostic accuracy for Timeless. Fig 1B showed that the AUC of Timeless achieved 0.941 in LUAD, indicating that Timeless had a high diagnostic accuracy. Survival curves with log-rank tests showed that LUAD patients with high Timeless expression had shorter survival times (Fig 1C).

### Suppression of Timeless impairs LUAD cell proliferation

**Fig 2:**
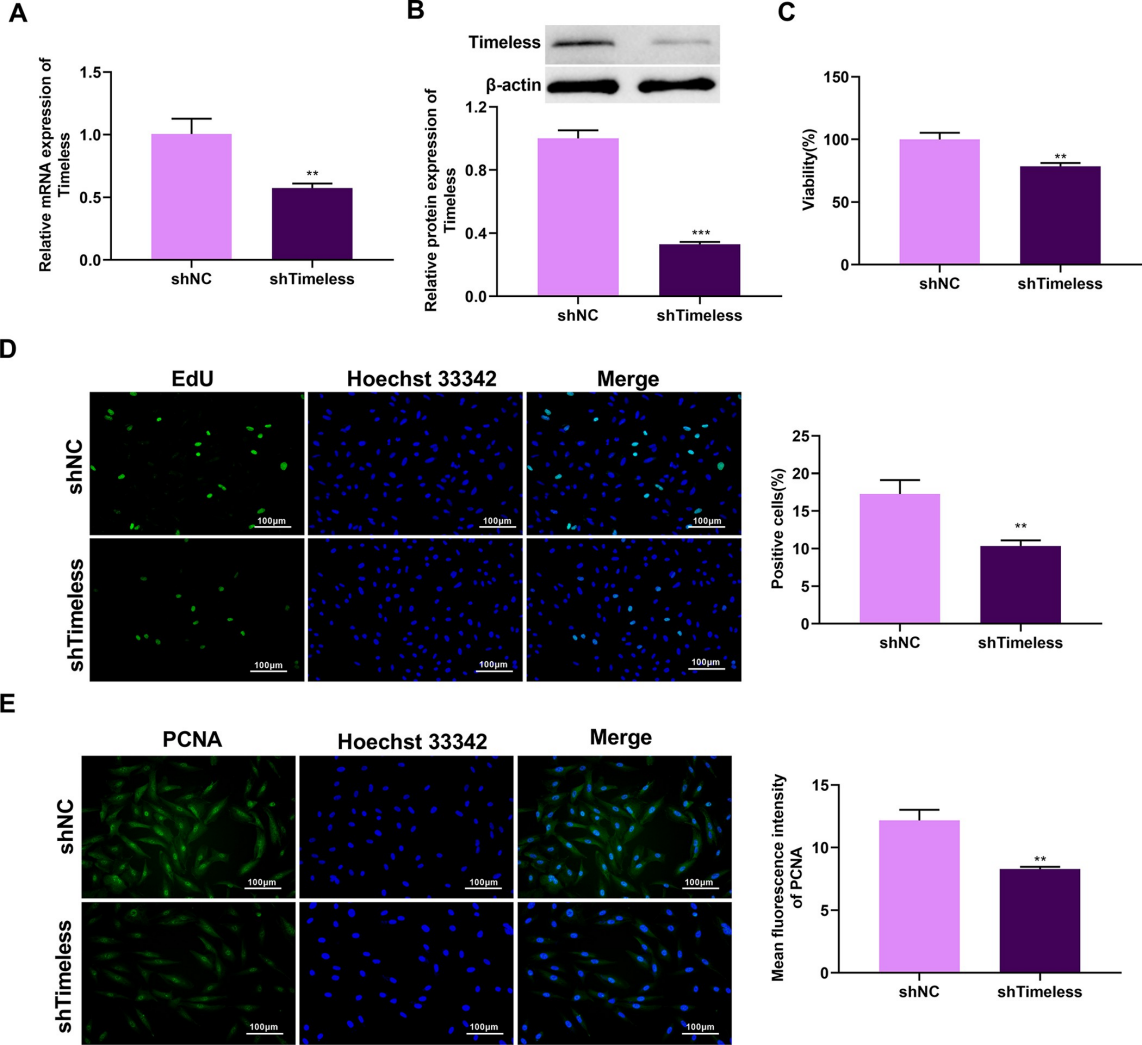
Inhibition of Timeless decreases A549 cell proliferation. (A) RT-qPCR analysis of the knockdown efficiency of lentiviral-mediated shTimeless on Timeless expression in A549 cells (n = 3). *p*-value based on a unpaired Student’s *t*-test. **p < 0.05 vs. shNC. (B) Western blotting analysis of the knockdown efficiency of lentiviral-mediated shTimeless on Timeless expression in A549 cells (n = 3). *p*-value based on a unpaired Student’s *t*-test. **p < 0.05 vs. shNC. (C) The proliferative capacity of Timeless-silenced A549 cells was evaluated by EdU assays (n = 3). *p*-value based on a unpaired Student’s *t*-test. **p < 0.05 vs. shNC. (D) The viability of Timeless-knockdown A549 cells was analyzed by CCK-8 assays (n = 3). *p*-value based on a unpaired Student’s *t*-test. **p < 0.05 vs. shNC. (E) Immunofluorescence staining was carried out to analyze PCNA protein levels in Timeless-knockdown A549 cells (n = 3). *p*-value based on a unpaired Student’s *t*-test. **p < 0.05 vs. shNC.

Given the upregulation of Timeless in LUAD, loss-of-function experiments were performed to ask the function of Timeless by transduction of shTimeless lentivirus into A549 and H1299 cells. Knockdown of Timeless by lentiviral-mediated shTimeless was confirmed by RT-qPCR (Fig 2A and S1A Fig) and western blot (Fig 2B). Indeed, the downregulation of Timeless was associated with a striking reduction in cell viability (Fig 2C and S1B Fig). Furthermore, the number of the EdU-positive LUAD cells was strongly diminished by Timeless silencing (Fig 2D and S1C Fig). A significant decrease in PCNA protein levels was also observed in Timeless-silenced A549 and H1299 cells (Fig 2E and S1D Fig). These data provided solid evidence to support the promoting effect of Timeless on A549 cell proliferation.

### Timeless is associated with PEM resistance of A549 cells

**Fig 3:**
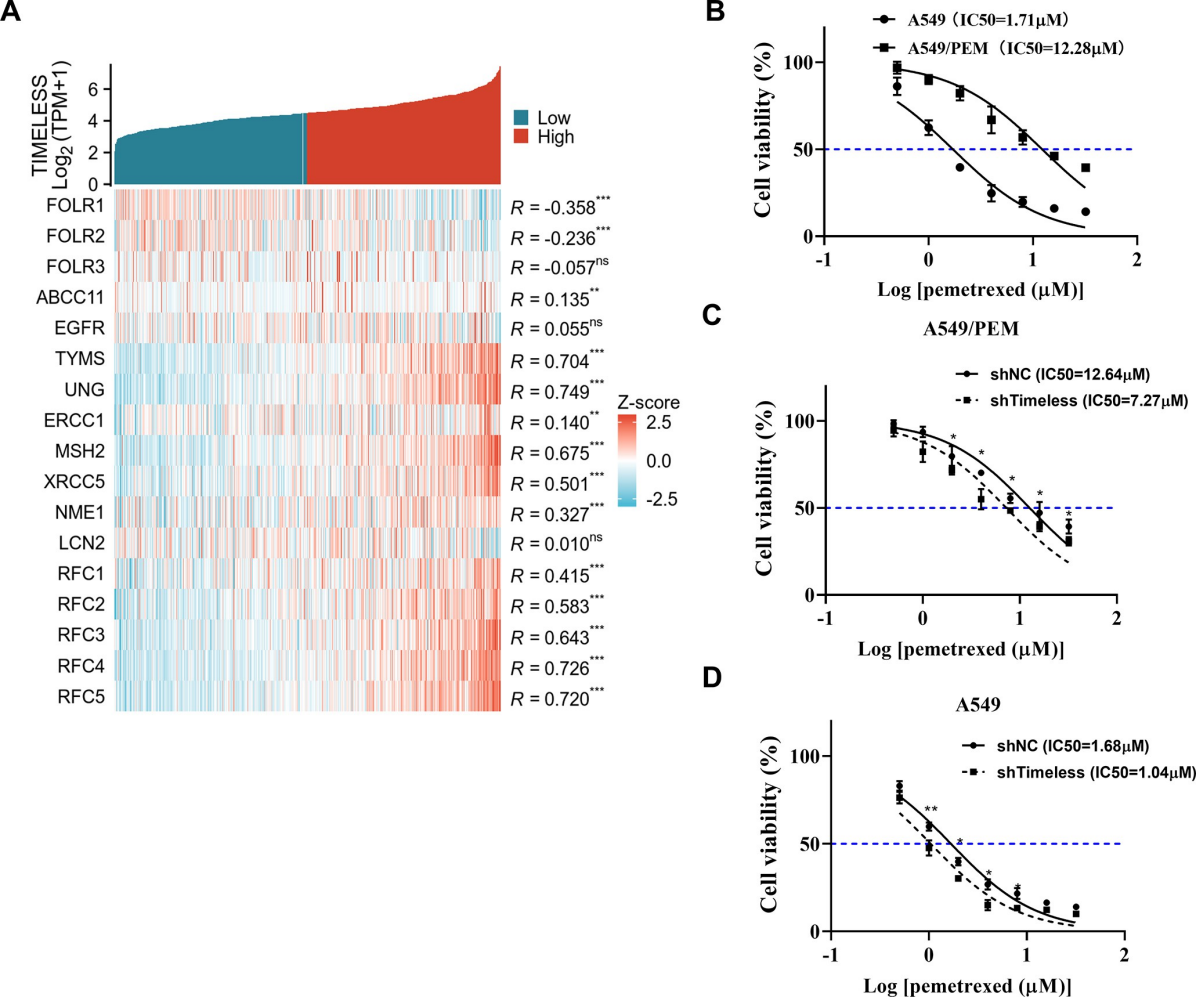
Timeless is associated with the resistance of LUAD to PEM. (A) The heatmap displayed transcripts related to Timeless expression in the RNA-seq data of TCGA-LUAD downloaded from the TCGA database. Statistical correlation data based on Spearman’s correlation analysis. ** *p* < 0.01, *** *p* < 0.001. ns: no significance. (B) Dose-response curves of A549/PEM and A549 cells exposed to different concentrations of PEM. *p*-value based on two-way ANOVA with Sidak’s multiple comparison tests (n = 3). (C and D) Drug sensitivity of PEM for A549/PEM and A549 transducted with shNC or shTimeless. *p*-value based on two-way ANOVA with Sidak’s multiple comparison tests (n = 3).

The RNAseq data of TCGA-LUAD downloaded from the TCGA database was analyzed to screen transcripts related to Timeless expression. As shown in Fig 3A, Timeless expression was correlated with FOLR1, FOLR2, ABCC11, TYMS, UNG, ERCC1, MSH2, XPCC5, NME1, RFC1, RFC2, RFC3, RFC4, and RFC5. Interestingly, some of these genes, such as FOLR1 [30], ABCC11 [31], TYMS [32], ERCC1 [33], and MSH2 [34], have been validated to be related to PEM resistance, suggesting that Timeless might be associated with the resistance of LUAD to PEM. We used PEM to treat A549 cells to confer PEM resistance. Compared with the parental cells, the IC50 value of A549/PEM cells was significantly increased, indicating that A549/PEM cells were successfully prepared (Fig 3B). In addition, A549/PEM cells showed high mRNA levels of Timeless than A549 parental controls (S2A Fig). Importantly, compared with the shNC group, knockdown of Timeless resulted in a significant decrease in the IC50 value of A549/PEM and A549 cells (Fig 3C and 3D). Collectively, these data indicate that to PEM-resistant A549 LUAD cells, depletion of Timeless is toxic.

### HPLC-MS/MS analysis identifies TFs that regulate Timeless expression in A549 cells

**Fig 4:**
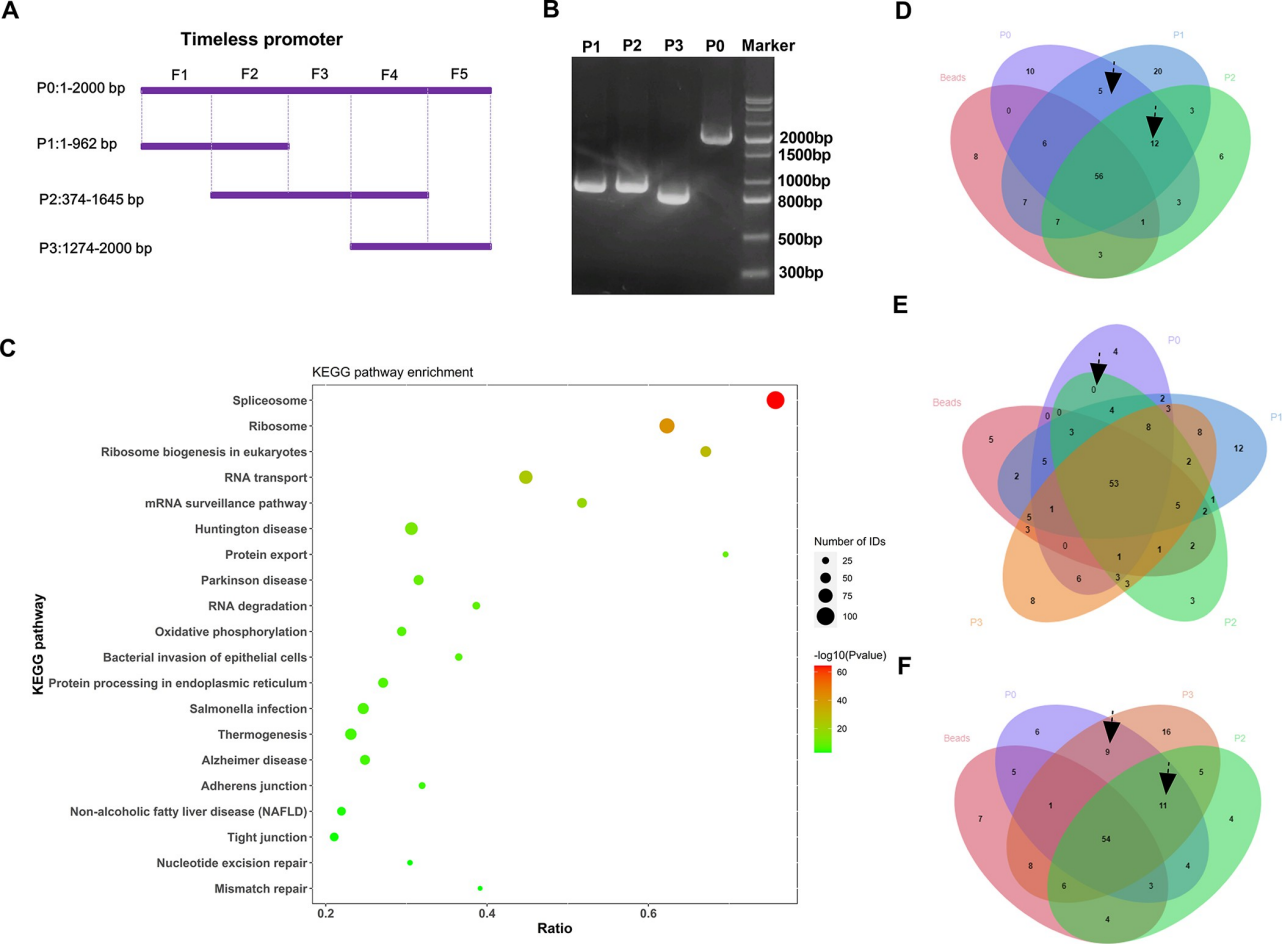
HPLC-MS/MS analysis identifies TFs that regulate Timeless expression in A549 cells. (A) Schematic model of the 4 biotin-labeled sequences (P0, P1, P2, and P3) and the five fragments (F1, F2, F3, F4, and F5) of the Timeless promoter. (B) The production of the 4 biotin-labeled sequences (P0, P1, P2, and P3) was validated by 1% agarose gel electrophoresis after amplification using the Timeless promoter sequence as a template. (C) KEGG functional enrichment analysis of proteins identified by HPLC-MS/MS analysis that could interact with biotin-labeled sequence P0. (D-F) Venn diagram showing the TFs pulled down by the five fragments (F1, F2, F3, F4, and F5).

To study the upstream regulators of Timeless in A549 cells, we conducted a qualitative proteomic analysis through HPLC-MS/MS analysis after DNA pull-down analysis to evaluate the TFs related to Timeless. PCR amplification was carried out with biotinylated primers to generate a biotinylated promoter with a full-length sequence (P0: 1–2000 bp) and 3 biotinylated promoters with truncated fragments (P1: 1–962 bp, P2: 374–1645 bp and P3: 1274–2000 bp) (Fig 4A). The amplification of the 4 biotinylated sequences was demonstrated by agarose gel electrophoresis (Fig 4B). The lysates of A549 cells were incubated with the four biotin-labeled promoter probes, respectively, and the collected precipitated proteins were analyzed using qualitative proteomic analysis. Their identified proteins were shown in S2 Table. The number of identified TFs was 93 (P0), 116 (P1), 91 (P2), and 110 (P3), respectively (S3 Table). We then conducted the KEGG pathway analysis to better understand the potential enrichment pathways of the identified proteins in the P0 group. As illustrated in Fig 4C, the spliceosome pathway had the highest enrichment rate in the top 20 signal pathways with significant changes. Importantly, five TFs were identified by the F1 fragment (1–373 bp, the TFs pulled down by P0 and P1 instead of P2 and beads) (Fig 4D); 12 TFs were identified with the F2 fragment (374–962 bp, the TFs pulled down by P0, P1 and P2 instead of beads) (Fig 4D); no TFs were identified by the F3 fragment (963–1273 bp, the TFs pulled down by P0 and P2 instead of P1, P3 and beads) (Fig 4E); a total of 11 TFs were identified by the F4 fragment (1274–1645 bp, the TFs pulled down by P0, P3 and P2 instead of beads) (Fig 4F); nine TFs were identified in the F5 fragment (1646–2000 bp, the TFs pulled down by P0 and P3 instead of P2 and beads) (Fig 4F). After eliminating duplicates, the 26 TFs identified by the 5 fragments (shown in S4 Table) were selected for subsequent analysis.

### Timeless is positively associated with HMGXB4, MAFG, MAZ, SP3, and ZNF384 that are related to LUAD prognosis

**Fig 5:**
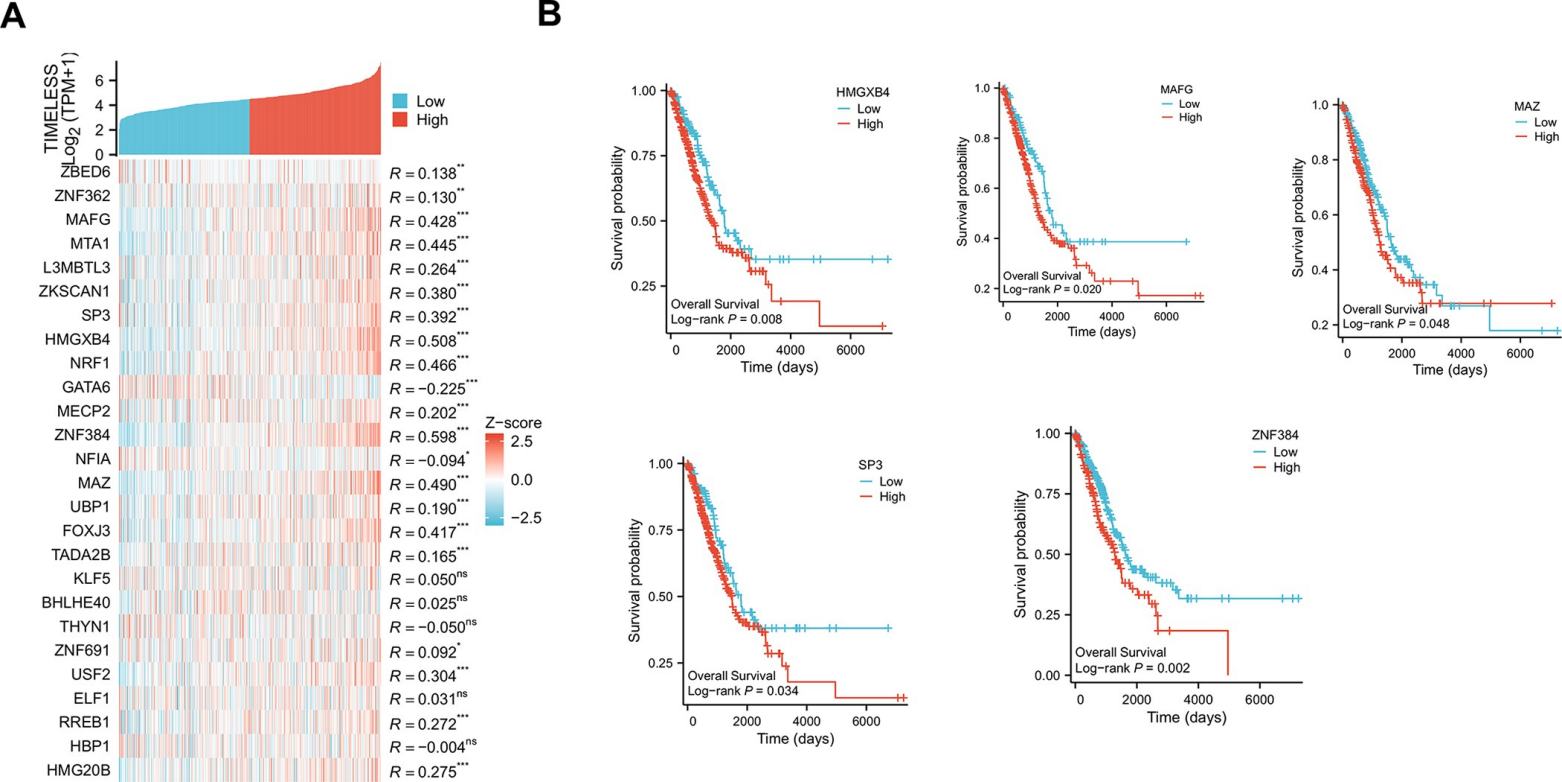
Association of Timeless and TFs in LUAD patients. (A) A heatmap of the correlation between Timeless and 26 identified TFs. * *p* < 0.05, ** *p* < 0.01, and *** *p* < 0.001. ns: no significance. (B) The prognostic significance of HMGXB4, MAFG, MAZ, SP3, or ZNF384 in patients with LUAD, according to the TCGA-LUAD database.

The correlations between Timeless and 26 identified TFs were analyzed. Among them, 9 TFs (MAFG, MTA1, ZKSCAN1, SP3, HMGXB4, NRF1, ZNF384, MAZ, and FOXJ3) were positively correlated with Timeless expression, with a correlation coefficient *R* > 0.35 (Fig 5A). Moreover, 5 TFs positively correlated with poor prognosis of LUAD (Fig 5B). Together, these results manifested the associations of Timeless expression and HMGXB4, MAFG, MAZ, SP3, and ZNF384 levels.

### SP3 positively regulates the transcription and expression of Timeless in LUAD cells

**Fig 6:**
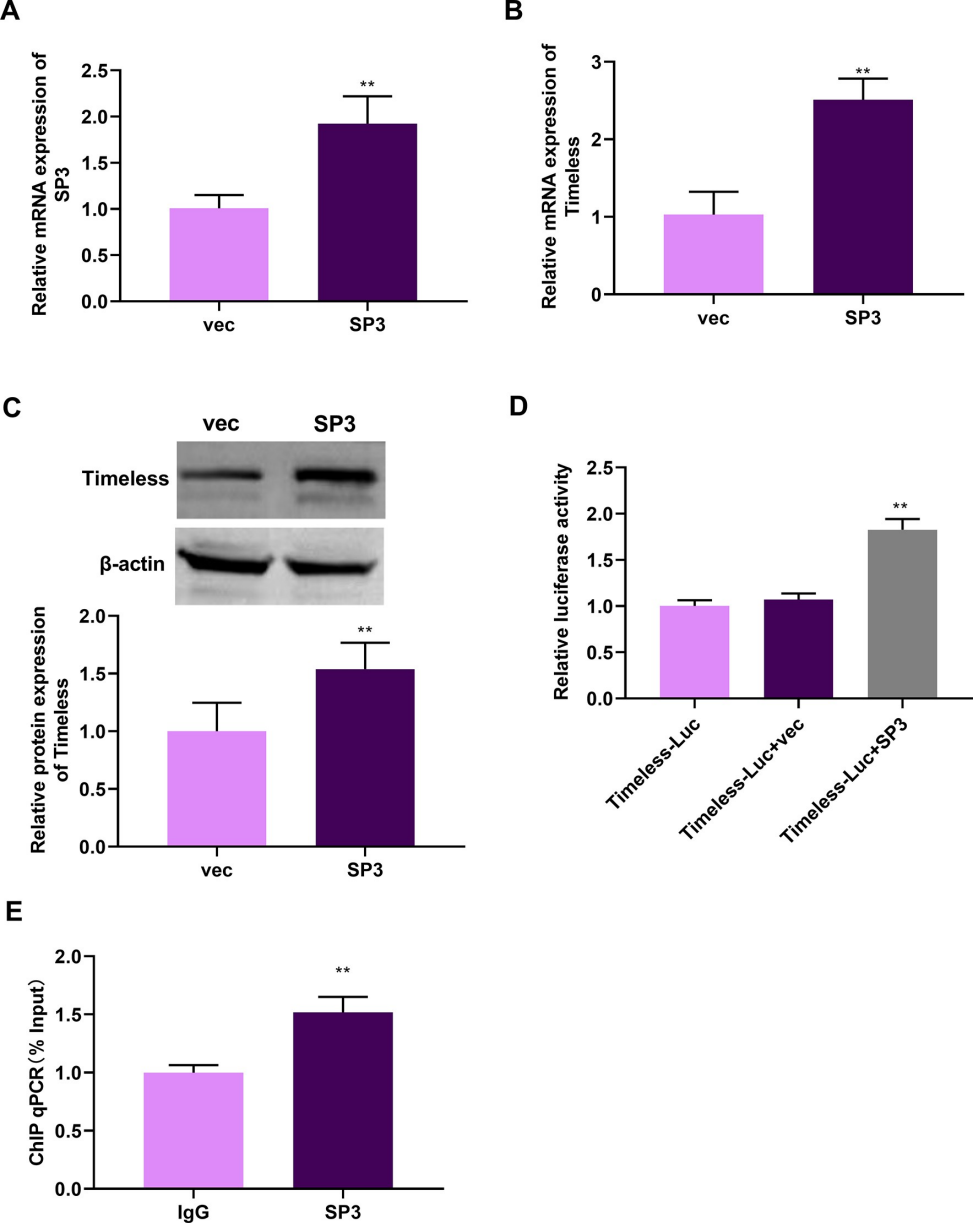
Timeless is positively regulated by Timeless in A549 cells. (A and B) Relative mRNA expression of SP3 and Timeless was evaluated by RT-qPCR in SP3-overexpressed A549 cells. *p*-value based on a unpaired Student’s *t*-test (n = 3). **p < 0.05 vs. vec. (C) Relative protein level of SP3 was analyzed by western blotting in SP3-overexpressed A549 cells. *p*-value based on a unpaired Student’s *t*-test (n = 3). **p < 0.05 vs. vec. (D) Effect of SP3 overexpression on the luciferase activity of the Timeless-Luc reporter in A549 cells. *p*-value based on one-way ANOVA followed by Tukey’s comparisons test (n = 3). (E) ChIP experiment was performed by incubating lysates of A549 cells with anti-IgG or anti-SP3 antibody, followed by the detection of the Timeless promoter containing the F2 fragment by PCR with specific primers. *p*-value based on a unpaired Student’s *t*-test (n = 3). **p < 0.05 vs. Timeless-Luc+vec.

SP3, which belongs to the SP family, is a non-oncogene addiction (NOA) gene and drug target in human cancers, including lung cancer [35]. Our data suggested that SP3 could bind to the Timeless promoter by the F2 fragment (374–962 bp) (S4 Table). To discuss the underlying mechanism between SP3 and Timeless, we obtained the pLV3-CMV-SP3 plasmid and transfected it into A549 and H1299 cells. Fig 6A and S1E Fig showed the overexpression efficiency of the pLV3-CMV-SP3 plasmid. We observed that Timeless expression at mRNA and protein levels was significantly elevated in SP3-overexpressed A549 cells, indicating that SP3 positively regulated Timeless expression (Fig 6B and 6C). Consistently, SP3 overexpression enhanced Timeless protein level in H1299 LUAD cells (S1F Fig). Luciferase reporter assays exhibited that SP3 overexpression significantly elevated the luciferase activity of the Timeless-Luc reporter relative to the vec group, suggesting that SP3 could enhance the transcription level of Timeless (Fig 6D and S1G Fig). Furthermore, ChIP-qPCR validated the direct relationship of SP3 and Timeless promoter in A549 and H1299 cells (Fig 6E and S1H Fig). Additionally, in line with Timeless mRNA expression, SP3 mRNA level was increased in A549/PEM cells compared with parental A549 cells (S2B Fig). Collectively, Timeless can be positively regulated by SP3 in A549 cells.

### SP3 affects cell growth by Timeless in LUAD cells

**Fig 7:**
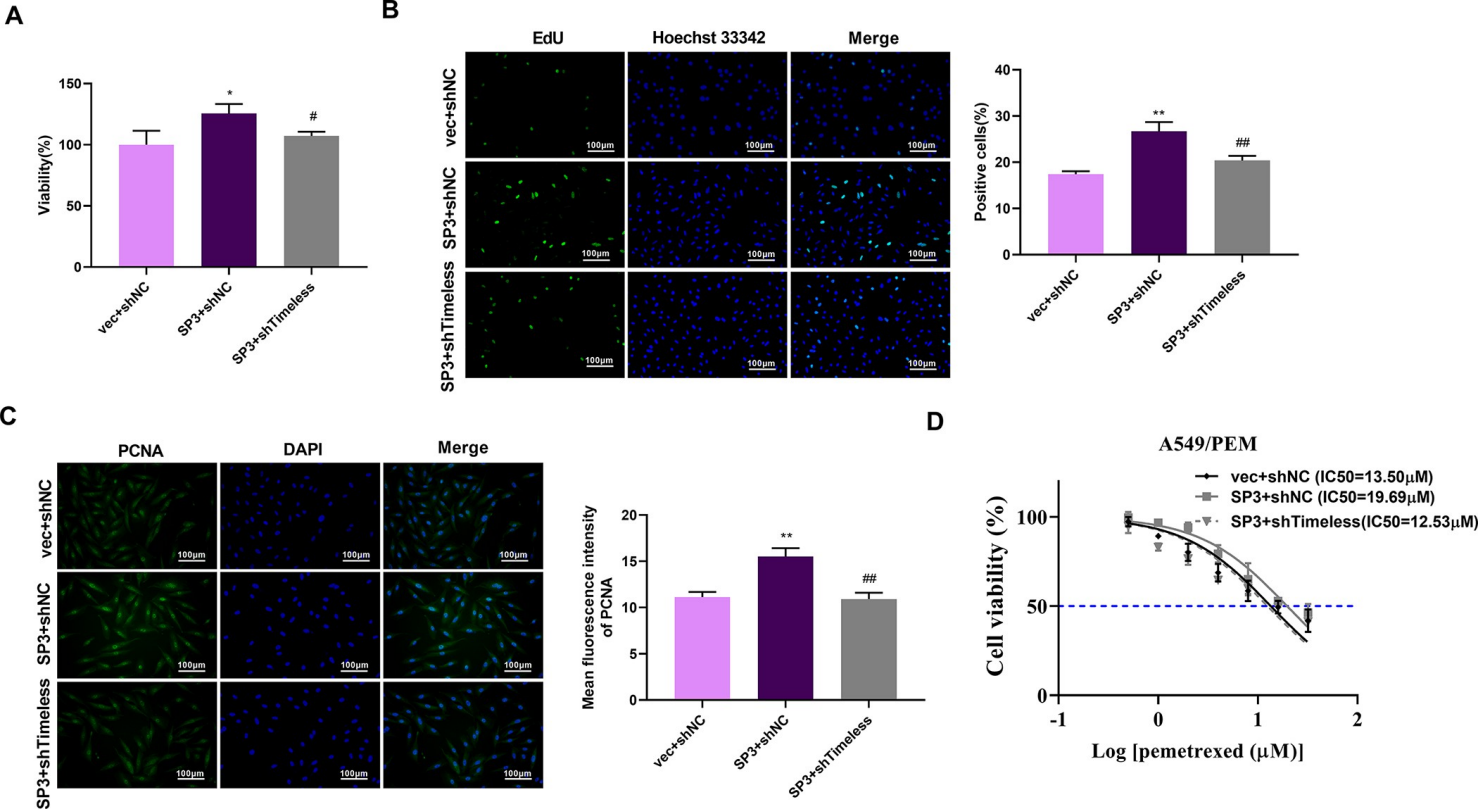
SP3 increases cell growth by Timeless in A549 cells. (A) Cell viability was determined by CCK-8 assays in A549 cells after transfection of vec+sh-NC, SP3+sh-NC, or SP3+shTimeless. *p*-value based on one-way ANOVA followed by Tukey’s comparisons test (n = 3). ** *p* < 0.01 vs. vec+sh-NC; ^##^ *p* < 0.01 vs. SP3+sh-NC. (B) Cell proliferation was detected by EdU staining in A549 cells transfected with the indicated vectors. *p*-value based on one-way ANOVA followed by Tukey’s comparisons test (n = 3). ** *p* < 0.01 vs. vec+sh-NC; ^##^ *p* < 0.01 vs. SP3+sh-NC. (C) Protein levels of PCNA were analyzed by immunofluorescence staining in A549 cells transfected with the indicated vectors. *p*-value based on one-way ANOVA followed by Tukey’s comparisons test (n = 3). ** *p* < 0.01 vs. vec+sh-NC; ^##^ *p* < 0.01 vs. SP3+sh-NC. (D) A549 cells transfected with the indicated vectors were treated with different concentrations of PEM. Drug sensitivity of PEM for A549 cells was assessed by CCK-8 assays. *p*-value based on two-way ANOVA with Sidak’s multiple comparison tests (n = 3).

Given these above data, we asked whether SP3 mediates LUAD cell proliferation by regulating Timeless expression. As expected, SP3 overexpression resulted in reinforced cell viability and proliferation (Fig 7A and 7B, S1I and S1J Fig). However, when SP3 overexpression was accompanied by Timeless silencing, the strengthening effect on A549 cell viability and proliferation was impaired (Fig 7A and 7B, S1I and S1J Fig). We further validated the promoting effect of SP3 on A549 and H1299 cell proliferation, as evidenced by an increase in PCNA protein levels upon SP3 overexpression. While, Timeless silencing significantly abated the elevation in PCNA protein levels caused by SP3 upregulation (Fig 7C and S1K Fig). We also wondered whether PEM resistance is associated with SP3 and Timeless. The results exhibited an elevation in the IC50 value of PEM in A549/PEM cells with overexpression of SP3, whereas downregulation of Timeless strongly reversed this elevation mediated by SP3 (Fig 7D). Taken together, these results suggested that SP3 enhances the growth of A549 cells by Timeless.

## Discussion

Timeless is an important component of the biological rhythm system. As a replication fork-related TF, Timeless can combine with expected interacting proteins to form stable complexes, thus affecting replication checkpoints and normal DNA replication [36]. Timeless has been reported to be upregulated in a variety of cancers, and it is associated with cancer occurrence and prognosis. For example, overexpression of Timeless is associated with low clinical survival and lymph node metastasis in early-stage cervical cancer [37] and cisplatin sensitivity in cervical cancer [20, 38] and nasopharyngeal carcinoma [19]. Analysis of the expression profile of NSCLC cell lines using microarray analysis showed that Timeless expression in NSCLC cell lines is increased by 3.7 times compared to normal lung cells [22], and reduction of Timeless in lung cancer cell lines H157 and H460 inhibits cell proliferation. Also, Timeless is significantly higher in NSCLC samples [39]. Timeless has a significant correlation with OS for NSCLC patients and is correlated with immune checkpoint and immune infiltration levels in NSCLC [21]. These data suggest that Timeless has an important role in NSCLC. However, its associated function and mechanism need to be further elucidated.

Here, the expression and clinical significance of Timeless were first analyzed by utilizing the TCGA-LUAD database. Analysis of the TCGA-LUAD database found that Timeless expression is significantly higher in LUAD patients than that of adjacent normal samples. Kaplan-Meier plots suggested that Timeless may be a potential prognostic marker for LUAD. ROC curve analysis identified Timeless as a very convincing biomarker for LUAD diagnosis. Functionally, we demonstrate that loss of Timeless inhibits LUAD cell growth. Our results were consistent with previous reports [21, 22, 39], which suggested that Timeless plays a promoting role in LUAD progression. To further understand the function of Timeless in carcinogenesis, we further identified related genes with Timeless using the TCGA-LUAD database. Considering the close expression associations of Timeless and PEM resistance-related genes, such as FOLR1 [30], ABCC11 [31], TYMS [32], ERCC1 [33], and MSH2 [34], we hypothesized that Timeless might be involved in LUAD resistance to PEM. Our data found that the knockdown of Timeless can result in a significant decrease in the IC50 value for PEM of A549/PEM cells. Overall, these data indicate that to PEM-resistant A549 LUAD cells, depletion of Timeless is toxic.

To further understand the potential regulators of Timeless, we identified TFs bound to Timeless using DNA pull-down technology with HPLC-MS/MS analysis. We here have identified a total of 26 TFs that can bind to the Timeless promoter. Considering the positive correlation with poor prognosis in LUAD, our data indicate the positive expression association of Timeless and HMGXB4, MAFG, MAZ, SP3, and ZNF384 levels. SP3 is involved in almost all cellular functions, including cell proliferation, apoptosis, and differentiation. SP3, a nuclear TF, is a transcription regulatory factor discovered after SP1. SP1 acts as an activator of transcription, while SP3 can act as both an activator and an inhibitor. Wang *et al*. pointed out that p53 participates in mediating the expression of Rab coupling protein in an SP1/3-dependent manner in lung cancer [35]. Tange *et al*. reported that SP3 mediates the transcription of β4GalT3 in A549 cells [40]. In addition, inhibition of expression of SP1/3-dependent ABCG2 sensitizes A549 cells to cisplatin [41]. A recent report revealed that Timeless interacts with SP1/c-Jun to promote c-Jun-mediated transcriptional induction of miR-5188, leading to the promotion of malignancy of breast cancer [27]. The report of Zhang *et al*. uncovered that SP1 upregulates ACER2 by interacting with SP1, leading to the promotion of cell growth and mitochondrial respiration [26]. Our data exhibited that SP3 positively regulates Timeless transcription. Moreover, SP3 promotes the proliferation of LUAD cells by activating Timeless expression. However, the clinical data in this study are based on public databases, which is limited to uncover the clinical significance of Timeless in LUAD. Moreover, the precise mechanism between SP3 and Timeless is only validated in A549 and H1299 LUAD cell lines *in vitro*, and more basic experiments are needed to verify their relationship in other LUAD cell lines in future work. Using Jaspar database (https://jaspar.genereg.net/) to predict the binding sites for SP3 within the Timeless promoter, we found four putative binding sequences (TGGGGAGGGTC, GTGAGTGTGGC, GGAGGTGGGTGGTGA and TTGGGTGGGGA) with the relative profile score threshold 85%. Moreover, the binding sites (GTGAGTGTGGC, GGAGGTGGGTGGTGA and TTGGGTGGGGA) were predicted within the F2 fragment, which supported our findings that SP3 can bind to the Timeless promoter by the F2 fragment (374–962 bp) (S4 Table). However, the current study is hampered by the lack of the demonstration of the binding sites, which is planned to be elucidated in future work. Because the effects of Timeless depletion and SP3 overexpression have not been tested in normal cells, it remains unclear whether their effects are neither cancer-specific nor PEM-resistance-specific, which is a big limitation in this study. The evaluation of potential therapeutic benefits and adverse side effects of these approaches should be warrant before clinical trials. Extensive analyses will be required to determine the safety of such approaches in various experimental models *in vitro* and *in vivo*.

In conclusion, the transcription of Timeless is regulated by TF SP3, thus promoting the proliferation of LUAD cells. Our study offers evidence that SP3 and Timeless play important roles in the progression of LUAD and may be potential therapeutic targets for LUAD.

## Supporting information

S1 Fig Timeless regulates H1299 cell proliferation by modulating SP3. * *p* < 0.05, ** *p* < 0.01, *** *p* < 0.001, ^#^ *p* < 0.05, ^##^ *p* < 0.01, ^###^ *p* < 0.01.
(TIF)

S2 Fig The mRNA levels of Timeless and SP3 in A549/PEM cells versus A549 cells. * *p* < 0.05, ** *p* < 0.01.
(TIF)

S1 Table Sequences of primers used in this study. (DOCX)

S2 Table The identified proteins based on the biotin-labeled sequences P0, P1, P2, and P3. (XLSX)

S3 Table The identified TFs in groups of sequences P0, P1, P2, and P3. (XLSX)

S4 Table The identified TFs in F1, F2, F3, F4 and F5 fragments. (XLSX)

S1 File (PDF)

S1 Raw images (PDF)

## Abbreviation

SP: Specificity protein
LUAD: lung adenocarcinoma
PEM: pemetrexed
TFs: transcription factors
KEGG: Kyoto Encyclopedia of Genes and Genomes
NSCLC: non-small cell lung cancer
OS: overall survival
ROC: receiver operating characteristic
FBS: fetal bovine serum
shRNA: short hairpin RNA
RT-qPCR: real time-quantitative PCR
EdU: 5-Ethynyl-2’deoxyuridine
CCK-8: Cell counting kit-8
OD: optical density
DAPI: 4’,6-diamidino-2-phenylindole
NOA: non-oncogene addiction
