# Supplementary figures and images for: SP3-induced Timeless transcription contributes to cell growth of lung adenocarcinoma cells
Source: PLoS One. 2024 Feb 14;19(2):e0298295. doi: 10.1371/journal.pone.0298295 (PMC10866488; doi:10.1371/journal.pone.0298295)

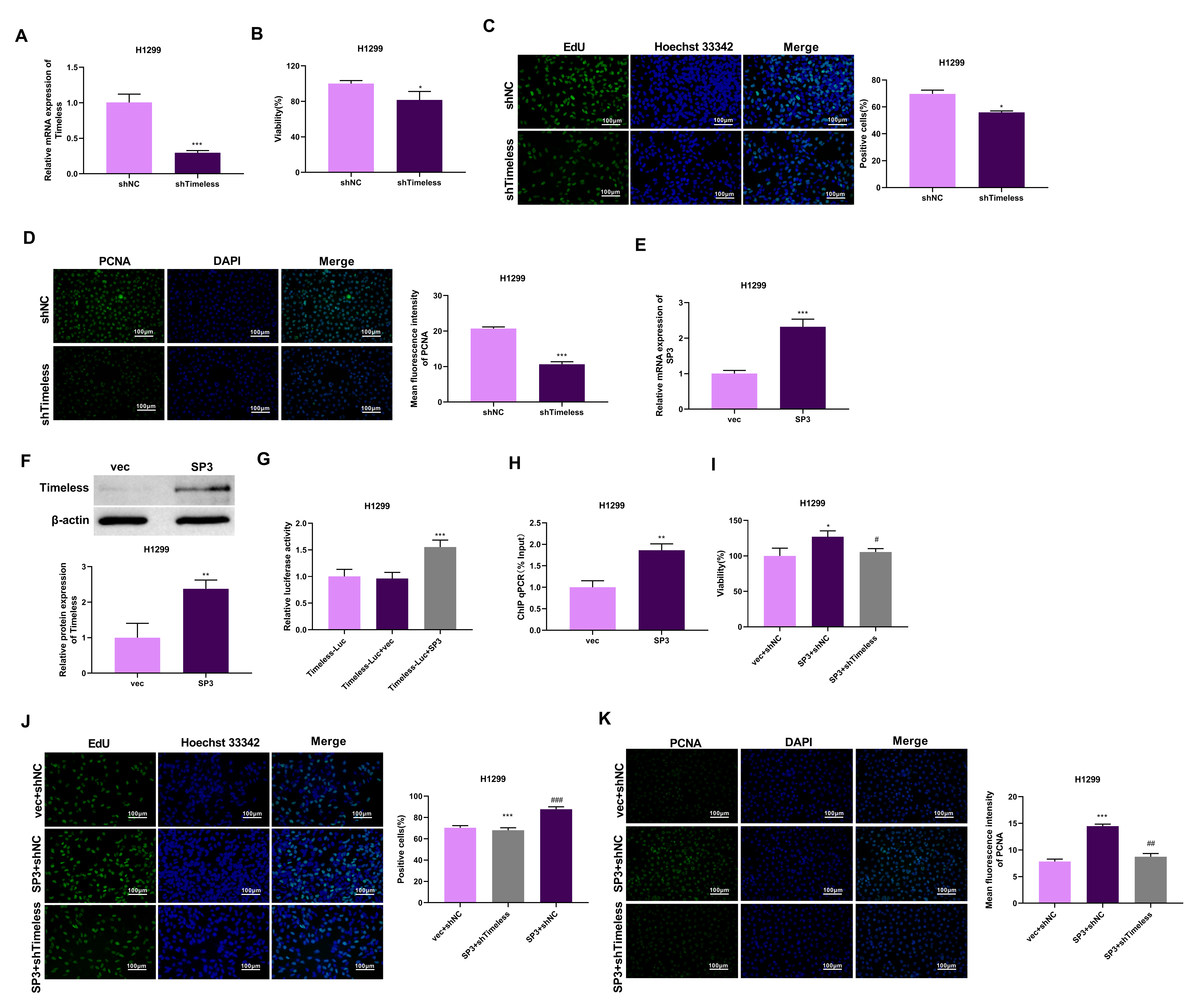

Supplement: S1 Fig — * p < 0.05, ** p < 0.01, *** p < 0.001, # p < 0.05, ## p < 0.01, ### p < 0.01. (TIF) [file pone.0298295.s001.tif]

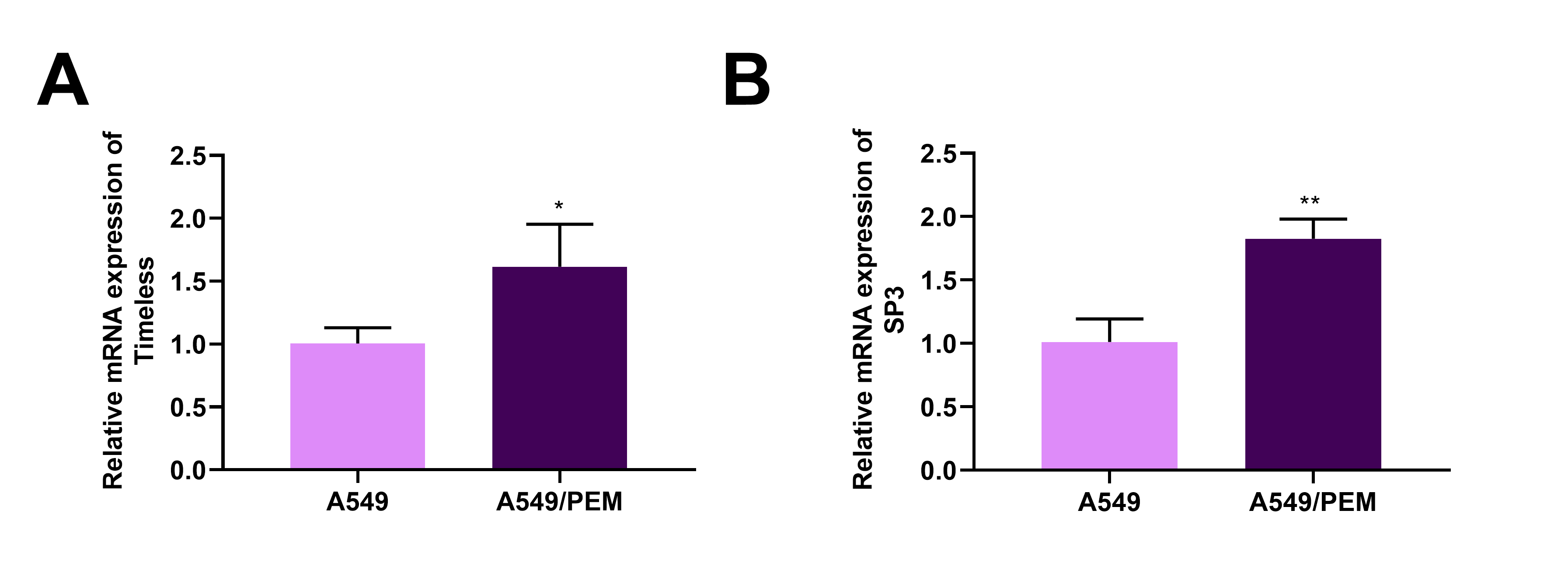

Supplement: S2 Fig — * p < 0.05, ** p < 0.01. (TIF) [file pone.0298295.s002.tif]
