# Supplementary material for: SP3-induced Timeless transcription contributes to cell growth of lung adenocarcinoma cells
Source: PLoS One. 2024 Feb 14;19(2):e0298295. doi: 10.1371/journal.pone.0298295 (PMC10866488; doi:10.1371/journal.pone.0298295)
Supplement: S1 Table — (DOCX) [file pone.0298295.s003.docx]

**Supplementary Table 1. Sequences of primers used in this study**

| Name | Sequence (5’-3’) |
| --- | --- |
| Timeless-F | TCTGATCCGCTATTTGAGGCA |
| Timeless-R | GGCAGAAGGTCGCTCTGTAG |
| GAPDH-F | GGGTGTGAACCATGAGAAGT |
| GAPDH-R | GACTGTGGTCATGAGTCCT |
| SP3-F | GGTCAAGTCCAGGTTCAGGG |
| SP3-R | CTGAGAACTGCCCGAGAGTC |
| Timeless-promoter-P0-F (5’-biotin) | AGCCTCCTGTACGACTTTCTTCTC |
| Timeless-promoter-P0-R (5’-biotin) | GGGGCGAGCCGGGCTGGTC |
| Timeless-promoter-P1-F (5’-biotin) | AGCCTCCTGTACGACTTTCTTCTC |
| Timeless-promoter-P1-R (5’-biotin) | AGGCTTGCCCAAGCTCTATTTA |
| Timeless-promoter-P2-F (5’-biotin) | TTACCCTGCTGCCAGTCAAGTG |
| Timeless-promoter-P2-R (5’-biotin) | GCAAGAGGCTCATAAAAGGAAG |
| Timeless-promoter-P3-F (5’-biotin) | GAAGCAAATCTCTTCTAACTCC |
| Timeless-promoter-P3-R (5’-biotin)  Timeless-promoter-F1 (ChIP-qPCR)  Timeless-promoter-R1 (ChIP-qPCR)  Timeless-promoter-F2 (ChIP-qPCR)  Timeless-promoter-R2 (ChIP-qPCR) | GGGGCGAGCCGGGCTGGTC  TTCTGGCCTTTACCCTGCTG  TCTGCCTCGTGTGAGGAGTA  GTGAGTGTGGCTGTGTCTGA  TACTGGGTCGAGGAAGGGAG |
