# Supplementary material for: SP3-induced Timeless transcription contributes to cell growth of lung adenocarcinoma cells
Source: PLoS One. 2024 Feb 14;19(2):e0298295. doi: 10.1371/journal.pone.0298295 (PMC10866488; doi:10.1371/journal.pone.0298295)

Marker：10kDa, 18kDa, 23kDa, 30kDa, 42kDa, 55kDa, 75kDa, 110kDa, 140kDa, 200kDa

Figure 2B-Timeless

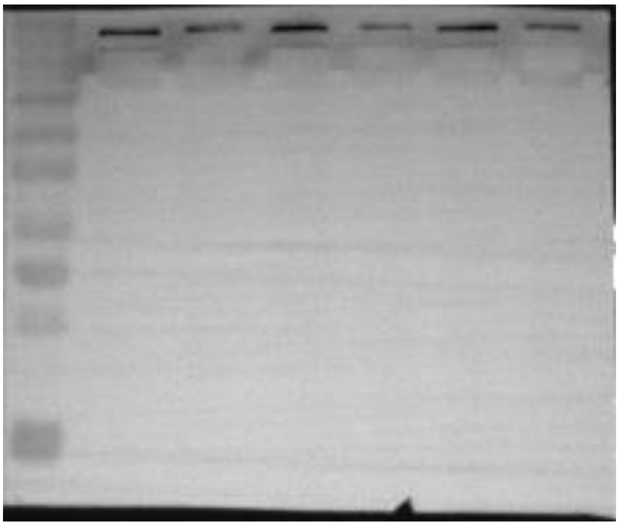

Figure 2B-β-actin

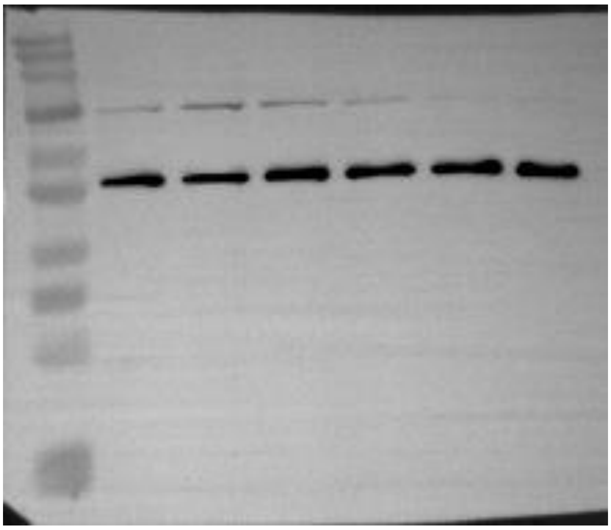

Figure 6C-Timeless

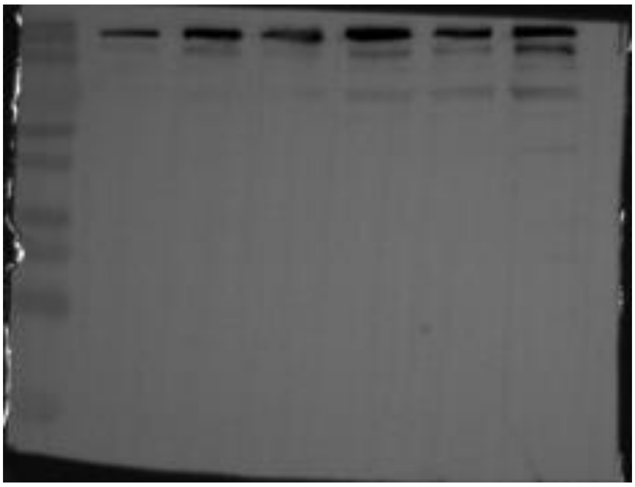

Figure 6C-β-actin

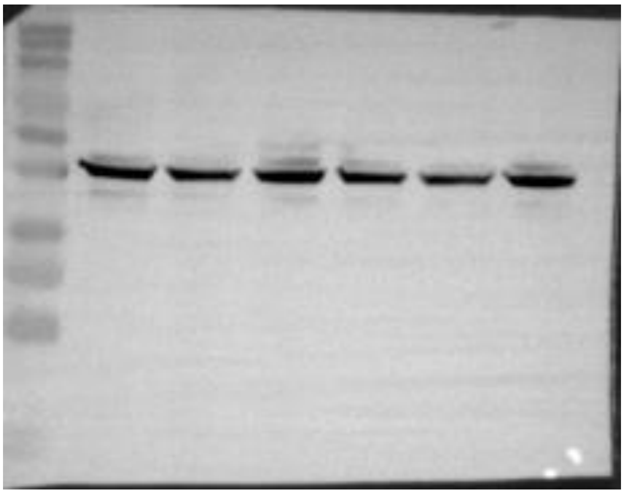

S1E Fig-Timeless

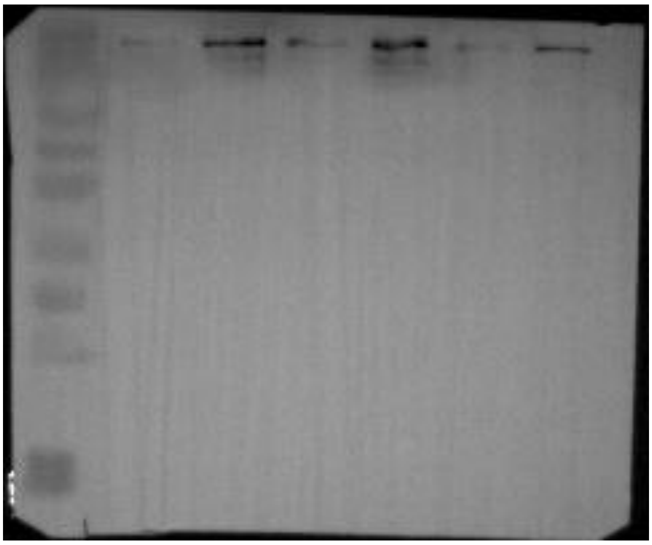

S1E Fig-β-actin

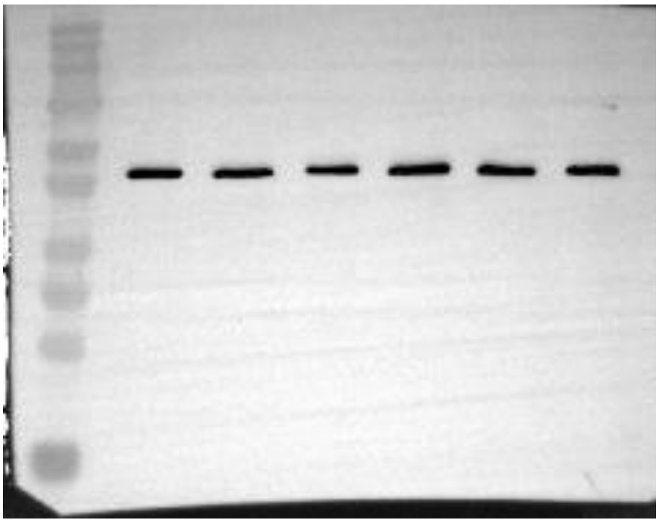

Supplement: S1 File — (PDF) [file pone.0298295.s007.pdf]
